# Parental perspectives on children’s social learning in four cultures

**DOI:** 10.3389/fpsyg.2026.1845262

**Published:** 2026-06-11

**Authors:** Jing Xu, Lara Wood, Claire Sutherland, Sarah Pope-Caldwell, Olivier Mboumba, Armel Elenga Ondaye, Grace Revenant Ngoma, Roger Ndenguele, Francy Kiabiya Ntamboudila, Sheina Lew-Levy

**Affiliations:** 1 Department of Anthropology, University of Washington, Seattle, WA, United States; 2 Department of Sociological and Psychological Sciences, Abertay University, Dundee, United Kingdom; 3 Department of Psychology, Georgia State University, Atlanta, GA, United States; 4 Lycée de Moukoundzi-Ngouaka, Brazzaville, Republic of Congo; 5 Faculté des lettres, arts et sciences humaines, Universite Marien Ngouabi, Brazzaville, Republic of Congo; 6 Ecole Nationale Supérieure d’Agronomie et de Foresterie, Universite Marien Ngouabi, Brazzaville, Republic of Congo; 7 Association des Jeunes pour l’Education a la Sauvegarde des Eléphants au Congo, Brazzaville, Republic of Congo; 8 Department of Psychology, Durham University, Durham, United Kingdom

**Keywords:** cross-cultural, cultural evolution (CE), developmental psychology, peer learning, social learning, parents, culture

## Abstract

Social learning is key to the accumulation and transmission of cultural knowledge that underpins human evolutionary success. Cultural context is critical for understanding the content, mechanisms, and pathways through which social learning occurs. Although parents constitute an important part of children’s social environment, parental perspectives on children’s social learning across cultures are understudied. Here, we worked with BaYaka and Bandongo living in the Republic of the Congo, Scots living in Tayside, United Kingdom, and Chinese Americans living in the Greater Seattle Area, United States, representing considerable diversity across ecological, economic, social, and cultural dimensions. 303 parents/guardians answered free-list and open-ended questions regarding what and how children should learn from peers and adults. Across cultures, parents consistently reported that children should learn from adults via mechanisms like imitation and teaching, but with peers via collaboration. In terms of learning content, BaYaka and Bandongo parents more often reported that children should learn tasks, whereas Scottish and Chinese American parents focused on qualities and values. Overall, parental reports reveal cross-cultural regularities in normative social learning mechanisms, while systematic differences in content highlight the importance of accounting for cultural context when studying the interaction between how, what, and from whom children learn.

## 1 Introduction

**Figure 1:**
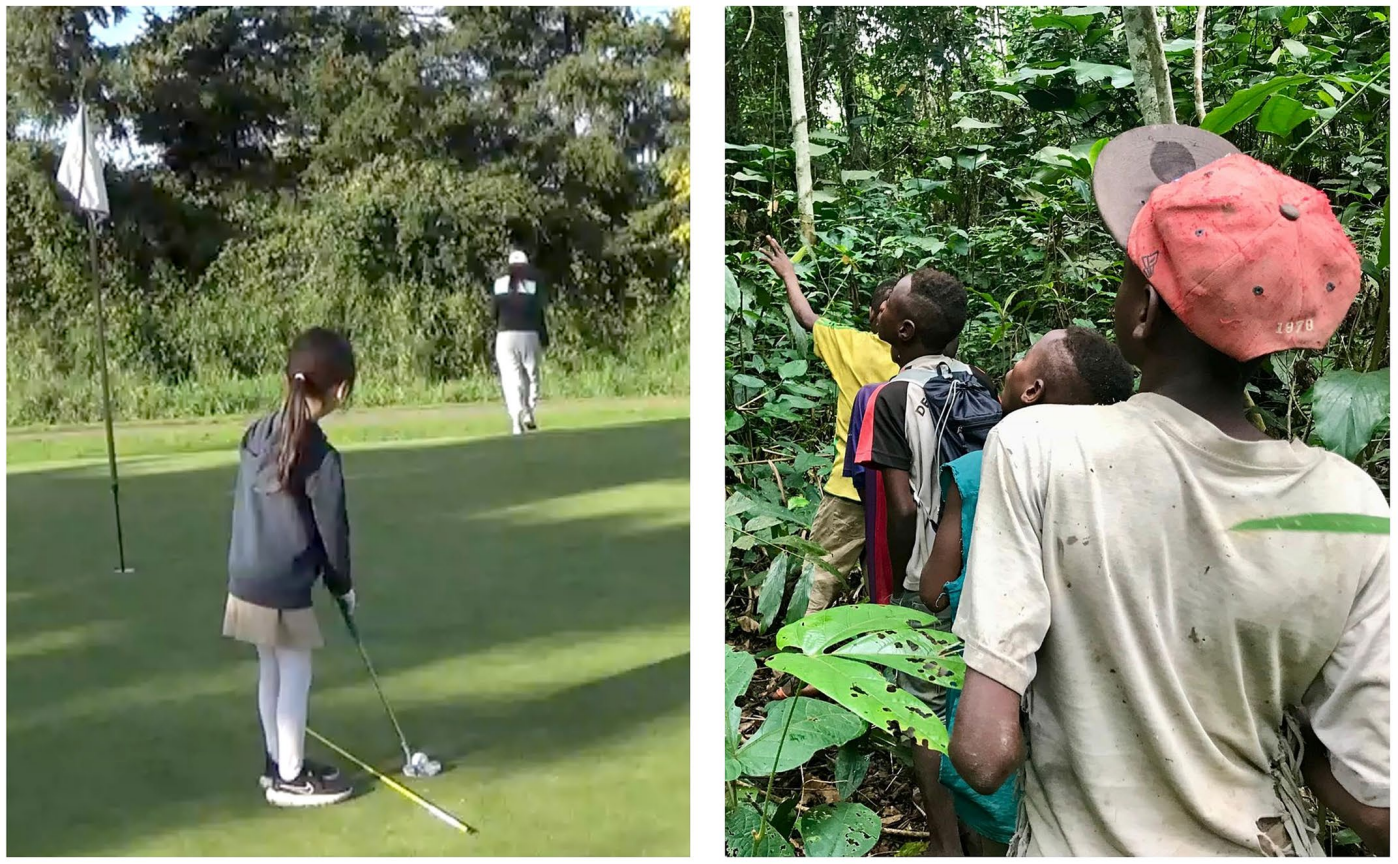
(Left) A young Chinese American child receives a private golf lesson. (Right) A group of Congolese BaYaka children forage autonomously in the forest.

Children learn a wealth of information from individuals in their social environment, which anthropologists and psychologists call social learning (Bandura, 1977; Boyd and Richerson, 1988). Social learning enables the accumulation and transmission of cultural knowledge that underpins human evolutionary success (Henrich, 2016). Children’s social environments, the types of knowledge and skills they acquire, and the ways social learning is organized differ dramatically across cultures (Lancy et al., 2011). Cultural context should play a primary, instead of secondary or peripheral role, in developing theoretical understanding about social learning and cultural evolution (Kline et al., 2018). Imagine these two scenarios documented as part of a large project on age-based imitation across cultures and contexts (Figure 1):

In an affluent suburb of Seattle, a young Chinese child is receiving a private golf lesson from a teacher accompanied by her parent, in preparation for a national tournament. In this setting, structured, adult-led, one-on-one instruction is a common form of children’s social learning even after children conclude a school day, and is often geared toward standardized competition and assessment.

In the forests of the Congo Basin, a group of mixed-age BaYaka children forage together without adult supervision. In many hunter-gatherer communities like the BaYaka (Lew-Levy et al., 2017), children are granted a great deal of autonomy, and they participate in subsistence activities at a young age, often learning together with peers.

These examples from cultural contexts with different ecological, economic, and social conditions illustrate three important dimensions in children’s social learning: “who”, adult-to-child (vertical or oblique transmission) or peer-to-peer (horizontal transmission); “what,” the content of learning; and “how”, the specific transmission mechanisms through which learning occurs. Recent scholarship has acknowledged that social learning is not fixed, but rather flexibly shaped by culture (Mesoudi et al., 2016). The “social learning of social learning” thus has a unique impact on culture and cultural evolution. Empirical work has started to systematically identify the similarities and differences along these dimensions of social learning behavior across diverse cultural contexts (Lew-Levy et al., 2023; Hewlett et al., 2024; Garfield and Lew-Levy, 2025). However, extant scholarship has less frequently examined parental perspectives on children’s social learning pathways, content, and mechanisms across cultures.

Anthropological research has long emphasized that parental beliefs about learning are culturally structured (LeVine and LeVine, 2016). “Parental ethnotheories” are defined as culturally constructed beliefs about children’s behavior and development, parenting, and family dynamics (Harkness and Super, 1996). These parental beliefs—reflecting broader cultural models of autonomy, relatedness, and learning—shape developmental expectations and parenting practices, which in turn affect child development pathways and outcomes (Keller, 2016).

To understand parental perspectives on social learning in diverse cultural settings, in the present study 303 parents/guardians (62% women, 38% men) answered free-list and open-ended questions regarding what and how children should learn from peers and adults in four communities: (1) BaYaka and (2) Bandongo living in the Republic of the Congo; (3) Scottish people living in Tayside, United Kingdom; and (4) Chinese Americans living in the Greater Seattle Area, a major hub for Chinese and East Asian immigrants in the United States. These study communities were selected because they vary considerably across ecological, economic, social, and cultural dimensions.

## 2 Study communities

Drawing from anthropological scholarship, here we conceptualize culture as a dynamic process embedded in ecology, political economy, and situated in history. In this view, processes of urbanization, market integration, formal education, migration, and global circulation of ideas continually shape socialization beliefs and practices, and individuals actively and agentively navigate these processes (Chapin and Xu, 2025). We expand on these dynamics in the following site descriptions.

In the Republic of the Congo, we worked in a multi-ethnic village shared by BaYaka foragers and Bandongo fisher-farmers. BaYaka rely on fishing, hunting with spears, trapping, horticulture, and collecting fruit, greens, mushrooms, nuts, seeds, and caterpillars for subsistence (Kitanishi, 1995). BaYaka children’s learning primarily occurs in autonomous multi-aged mixed-gender peer groups, where children teach each other, play together, and participate in foraging (Lew-Levy et al., 2019, 2020; Salali et al., 2019). BaYaka spend 3–6 months living in forest camps, and the remainder of the year in the study village, where they often work for Bandongo (Boyette, 2022; Kandza et al., 2024). Bandongo primarily subsist on fishing, horticulture, and hunting. Bandongo parents play a normative role in child socialization (Boyette et al., 2018), though children spend considerable time in same-gender peer groups participating in household chores and subsistence activities. Most BaYaka and Bandongo parents in the study village have some primary education. There is a primary school in the study village, and while parents from both communities encourage their children to attend, BaYaka face more barriers to participation in formal education than Bandongo (Bombjaková et al., 2020; Ninkova et al., 2025). The village is currently minimally market integrated, and only accessible by boat or walking (Lew-Levy et al., 2025).

In Scotland, the research was completed within the Tayside region which has a mix of urban and rural communities including two cities. Typical family structures include nuclear, single-parent, and blended families. The schooling system is overseen by the Scottish Government, with local education authorities managing day-to-day operations. Formal education is compulsory and fully funded for five- to 16-year-olds, with additional funding of nursery school for three-year-olds and post-16 education, including undergraduate university fees. Children are not generally expected to contribute to the household economy, focusing instead on formal education and qualifications. The schooling system is overseen by the Scottish Government, with local education authorities managing day-to-day operations. Children are legally considered adults at age 16 with most adult rights and responsibilities at this age.

The Greater Seattle Area is a major hub for Chinese immigrants in the United States, both historically since the 1850s and during today’s waves of educational and professional immigration. This area has a high median-income by United States national standard, largely due to its Information Technology economy and residents’ high educational credentials. Participating families included a variety of professional backgrounds including stay-at-home parents, socio-economic statuses, and household types including nuclear, extended, and single-parent households. Parents, especially mothers, are highly involved in childcare, and children live a busy and structured life of schooling (including home schooling families) and various extra-curricular activities. Therefore, children have ample opportunities to socialize with parents, peers, and other adults such as teachers, extra-curricular instructors, family friends, and relatives.

## 3 Methods

### 3.1 Approvals

We obtained ethical approval from the Department of Psychology at Durham University (no. 077); Psychology at Abertay University (EMS8703); and the Institutional Review Board at the University of Washington (STUDY00019988). The Institut National de Recherche en Sciences Sociales et Humaines (INRSSH) provided research permission for data collection in the Republic of the Congo. In Scotland and the United States, we obtained written consent from participants. In the Republic of the Congo, we obtained community consent before the start of research, and verbal consent from participants.

### 3.2 Data collection

Data for the present study was collected between May 2024 and September 2025 as part of a larger project on age-based imitation across cultures and contexts (Wood et al., n.d.; Visine et al., n.d.; Lew-Levy et al., 2026a). See Table 1 for sample characteristics. Directly following a forced-choice parenting norms questionnaire (discussed in Lew-Levy et al., 2026a), parents were asked two pairs of ethnographic questions. In the first pair, parents were asked to make a list in response to each of the following questions:

- What should children learn from other children?
- What should children learn from adults?

**Table 1:** Sample characteristics.

Study community	*N* (% women)	Peer-focused free-list		Adult-focused free-list	
		Mean No. responses	Max No. responses	Mean No. responses	Max No. responses
Bandongo	88 (48.9)	5.77	13	5.45	12
BaYaka	67 (62.7)	5	8	4.69	9
Scots	95 (60)	4.53	13	6.24	22
Chinese Americans	53 (84.9)	3.02	7	3.08	7

Parents were also asked to provide a narrative response for each of the following questions:

- How should children learn from other children?
- How should children learn from adults?

In Congo, these questions were asked verbally by researchers and recorded on paper. In Scotland and the United States, participants responded to these questions on Qualtrics, an online survey tool. Questions were forward- and back-translated from English or French by speakers fluent in Mandarin (Chinese Americans), Lingala (Bandongo), and Yaka (BaYaka). Chinese American participants could choose to answer questions in either Mandarin or English.

### 3.3 “What”: free lists

To harmonize across the free list “what” questions, SLL organized responses into representative codes. These codes were reviewed by JX and further refined into 27 categories (see Supplementary materials for definitions). 10% of each study community’s responses were also coded by SPC. Interrater reliability was high (*n* = 290, 85% agreement; Gwet’s AC1 = 0.89, 95% CI [0.85, 0.92]).

Following cultural consensus theory, we used the AnthroTool package (Purzycki and Jamieson-Lane, 2017) in R (R Core Team, 2025) to compute Smith’s Salience for each study community. Smith’s Salience is the sum of the percentile ordered rank position of an item within each list divided by the total number of lists (Smith, 1993). As it accounts for both frequency and rank, Smith’s salience provides an “overall notion of domain item salience: how often and how early culturally competent informants name the items that comprise a domain’s most important elements” (Smith, 1993, p. 3). Because we recoded items, some codes appear more than once in a list. Since repeated items can inflate Smith’s Salience values, we considered only the first instance a participant listed an item and disregarded any later mentions using the MAX function in AnthroTools.

### 3.4 “How”: open questions

We (JX, LW, SLL) conducted an abductive qualitative content analysis (Thompson, 2022), which then guided the development of quantitative categorical variables. Specifically, we first read through the responses to the “how” questions to get a sense of similarities and differences in the types of teaching and learning reported by participants across the study communities. Guided by discussion of our observations and the prior literature on cultural learning (Tomasello et al., 1993), we generated three non-mutually-exclusive categories: self-initiated learning, other-initiated learning, and collaborative learning. To validate these categories, 10% of each study community’s responses were also coded by SPC. Interrater reliability was high for all categories (*n* = 180, 87% agreement; Gwet’s AC1 = 0.82, 95% CI [0.75, 0.89]).

*Self-initiated learning* was operationalized as a learner independently seeking knowledge from another individual. This included observational learning (Paradise and Rogoff, 2009), indexed by words such as observing, listening, watching, approaching, evaluating. Instances in which a learner was *called* with the intention to facilitate their observation of a learning task was *not* coded as self-initiated, but rather other-initiated (see below). This category also included imitative learning, defined by Tomasello et al. (1993, p. 497) as “reproducing the adult’s actual behavioral strategies in their appropriate functional contexts,” and indexed by words such as imitation, following, copying, learning from example, repeating what others do, emulative play, mirroring. Other aspects of self-initiated learning included asking questions, asking for help, and reading as a way of acquiring knowledge.

*Other-initiated learning* was operationalized as another individual seeking to transmit knowledge to a learner. This included explicit and overt teaching (Kline, 2017), indexed by words such as teaching, explaining, instructing, demonstrating, showing, giving advice, coaching, directing. We also included instances where others seek to more subtly teach by providing opportunities for learning or by setting up an environment conducive to learning, as indexed by words such as scaffolding, mentoring, setting the example, modelling, encouraging, supporting, structuring, calling (e.g., to observe), assigning tasks.

*Collaborative learning* is defined by Tomasello et al. (1993, p. 500) as occurring when “neither interactant is an authority or expert; the intersubjectivity is symmetrical.” This included learning by being with others (Lave and Wenger, 1991), as indexed by words like spending time together, shared experience, interacting, socializing, mutual interest. This category also included participating in activities with others (Paradise and Rogoff, 2009), as indexed by words such as joining in, engaging, working together, helping, taking turns, playing. Finally, collaborative learning also involved communication (Köymen and Tomasello, 2020; Langenhoff et al., 2025), as indexed by words such as discussion, knowledge exchange, sharing ideas. A subset of communicative acts involved negotiation, as indexed by words such as arguing, debate, disagreement, competition. If a learner does an activity *with* someone else *in order to* learn/be taught, this was coded as self-initiated rather than collaborative learning.

These data were analyzed using a Bayesian multilevel logistic regression model implemented in *brms* (Bürkner, 2018) in R. The outcome variable was binary, reflecting the presence/absence of each learning category (i.e., three observations per participant per learning model). The statistical model included fixed effects for learning model (peer, adult), learning category (self-initiated learning, other-initiated learning, collaborative learning), and study community, and all interactions among these predictors. To account for repeated observations, we included random effects for participants. Specifically, the statistical model contained varying intercepts for participants and varying slopes for learning model, learning category, and their interaction. In other words, observations were nested within participants, allowing the effects of learning model and learning category to vary across individuals. We specified weakly informative priors. The statistical model was fit on four chains of 10,000 iterations each.

We further used a computational text analysis method, Term Frequency-Inverse Document Frequency (TF-IDF), implemented in the Python library scikit-learn (Pedregosa et al., 2011), to identify distinct linguistic patterns of participants’ responses to the two open-ended questions across the four study communities. TF-IDF is a widely used text-mining statistical method that evaluates the importance of a word within a document relative to a collection, through multiplying how often the word appears in that document (TF) by how unique it is across the entire collection (IDF). In our study, individual responses were first aggregated into eight corpus-level documents—organized by study community and peer- and adult-focused questions—to mitigate the sparsity of short text and allow for a robust comparison of group-specific lexicons. These texts were processed using a stemming algorithm that cuts word endings, e.g., turning “observation” and “observe” both into “observ,” to ensure that variants of the same root form are treated as a single unit, then restored the high-frequency original words in the results. We also added a custom list of words that were not included in the analysis (“children”, “child”, “kid”, “kids”, “learn”, “adult”, “adults”, “parent”, “parents”, “peer”) beyond the standard English stop word list in the scikit-learn package. TF-IDF analysis allows researchers to extract salient themes from unstructured qualitative data, and is particularly effective for cross-cultural analysis as it suppresses common “noise” terms and prioritizes words and phrases that are unique to one group compared to the others (Manning et al., 2008).

## 4 Results

### 4.1 “What”: free lists

**Figure 2:**
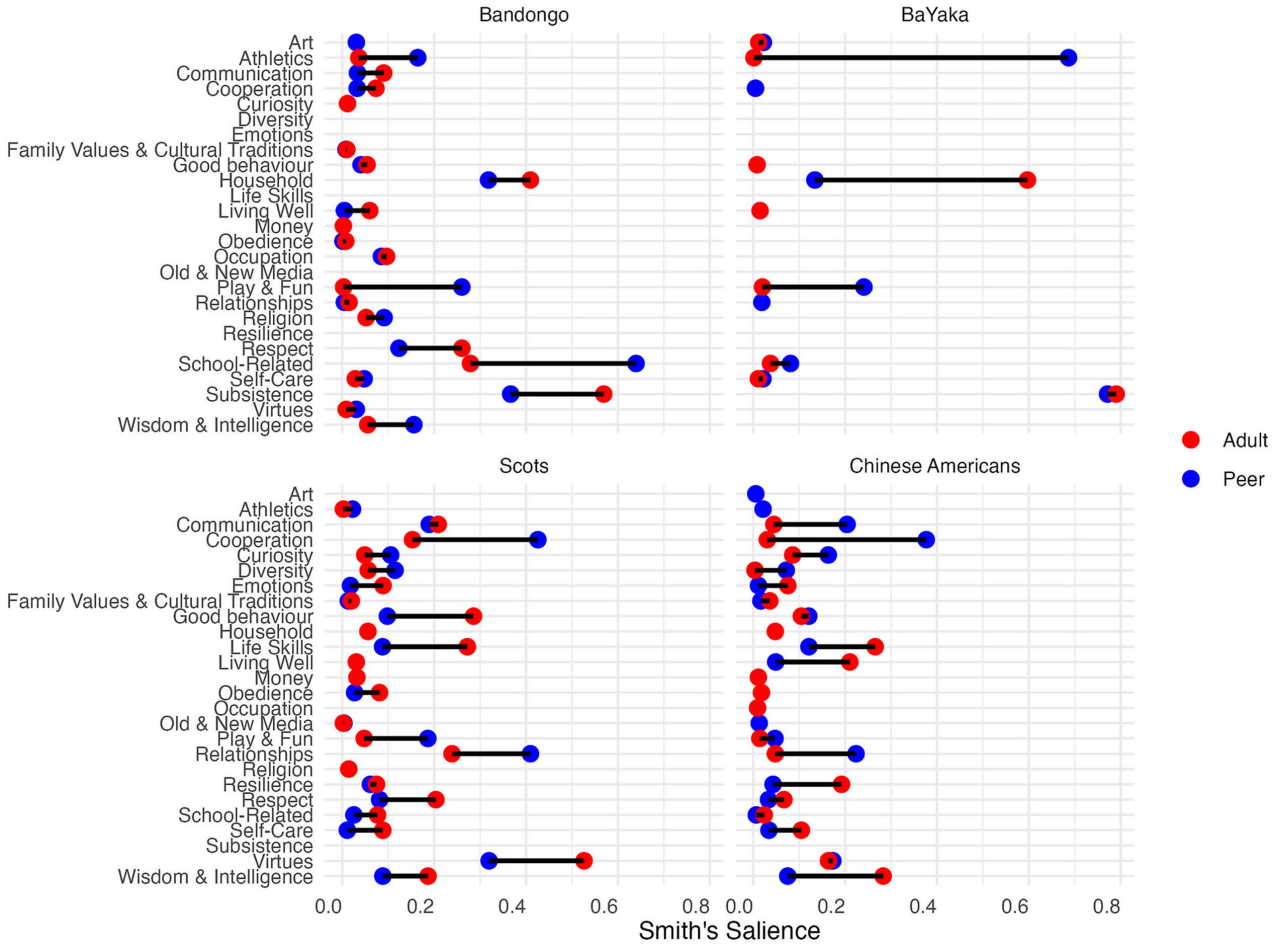
Smith’s salience values for peer- and adult-focused free lists, *N* = 298.

Figure 2 shows free list responses for peer-focused and adult-focused questions (see also Supplementary Table S1). Salience was overall high for Bandongo and BaYaka, reflecting stronger convergence on item importance; in comparison, salience was most distributed for Chinese Americans. For Bandongo, learning School-Related knowledge (e.g., reading, writing) and Play and Fun (e.g., games, having fun) were salient items learned from peers. Subsistence activities, Household tasks, and Respect (e.g., respecting elders) were salient items learned from adults. For BaYaka, Subsistence activities (e.g., hunting, fishing) was the most salient category, with a small preference for adult learning; Household tasks (e.g., cooking) was a salient item more strongly learned from adults. Athletics (e.g., swimming) and Play and Fun were salient items learned from peers. Among Scots, Virtues (e.g., kindness), Good Behavior (e.g., being courteous), and Life Skills (e.g., time management) were salient items learned from adults, whereas Cooperation (e.g., teamwork) and Relationships (e.g., setting boundaries, social skills) were salient items learned from peers. Similarly, for Chinese Americans, Cooperation and Relationships were salient for peer learning, whereas Wisdom & Intelligence (e.g., decision making), Life Skills, and Living Well (e.g., happiness) were the most salient items learned from adults.

### 4.2 “How”: open questions

**Figure 3:**
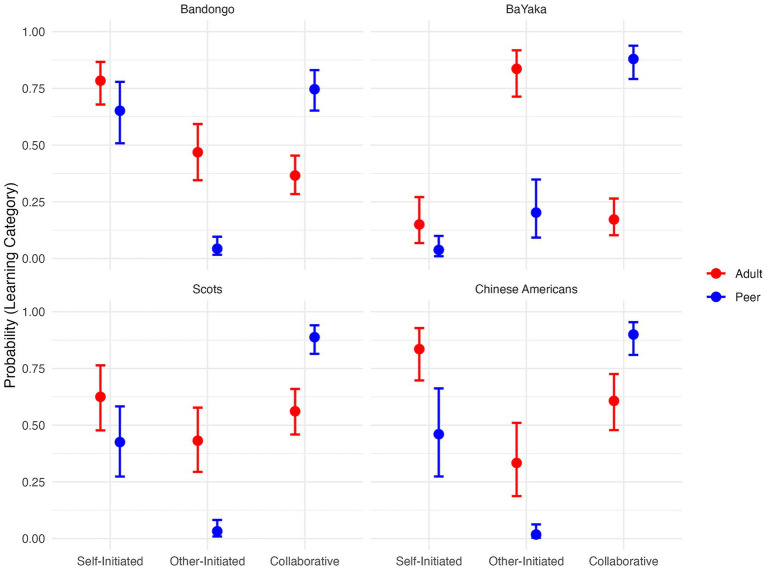
Results from the multilevel logistic regression model showing the probability of a learning category per target learning model (peer or adult) and study community. Points reflect mean estimates with 95% credible intervals, *N* = 294.

Table 2 gives representative responses for each study community and learning category. Statistical analysis (Figure 3; Supplementary Table S2) revealed consistent cross-cultural trends: Self- and Other-Initiated learning were more commonly reported for adult-focused learning, while Collaborative learning was more common for peer-focused learning. These patterns were confirmed by strong (i.e., 95% HPDIs did not cross 0) adult – peer contrasts across all comparisons (Supplementary Table S3), except for Bandongo Self-Initiated learning reports, which nonetheless trended in the same direction.

**Table 2:** Representative responses regarding how children should learn from peers/adults.

Study community	Self-initiated	Other-initiated	Collaborative
Bandongo	“The children must observe with attention while the other children work to benefit from their knowledge”	“Children must apply the advice given to them by parents”	“Children must be friends and must collaborate together, children acquire knowledge through mutual help”
BaYaka	“Observation comes before in all things, because without eyes we cannot learn”	“Parents teach children how to cook, draw water, wash dishes and how to swim”	“If the child walks with others, s/he can stop fighting and take intelligence from the others”
Scots	“Listening to adults, asking questions, observing them and copying”	“Adults need to model the behavior they expect their children to follow”	“Peer learning is most effective when it occurs in supportive environments where children can collaborate, explore, and build relationships”
Chinese Americans	“Observing peers’ behavior that they are interested in and imitating it, at a certain age adding their own judgment about whether it is correct and suitable for themselves”	“Follow adults’ instructions after understanding them”	“Playing and living together”

**Figure 4:**
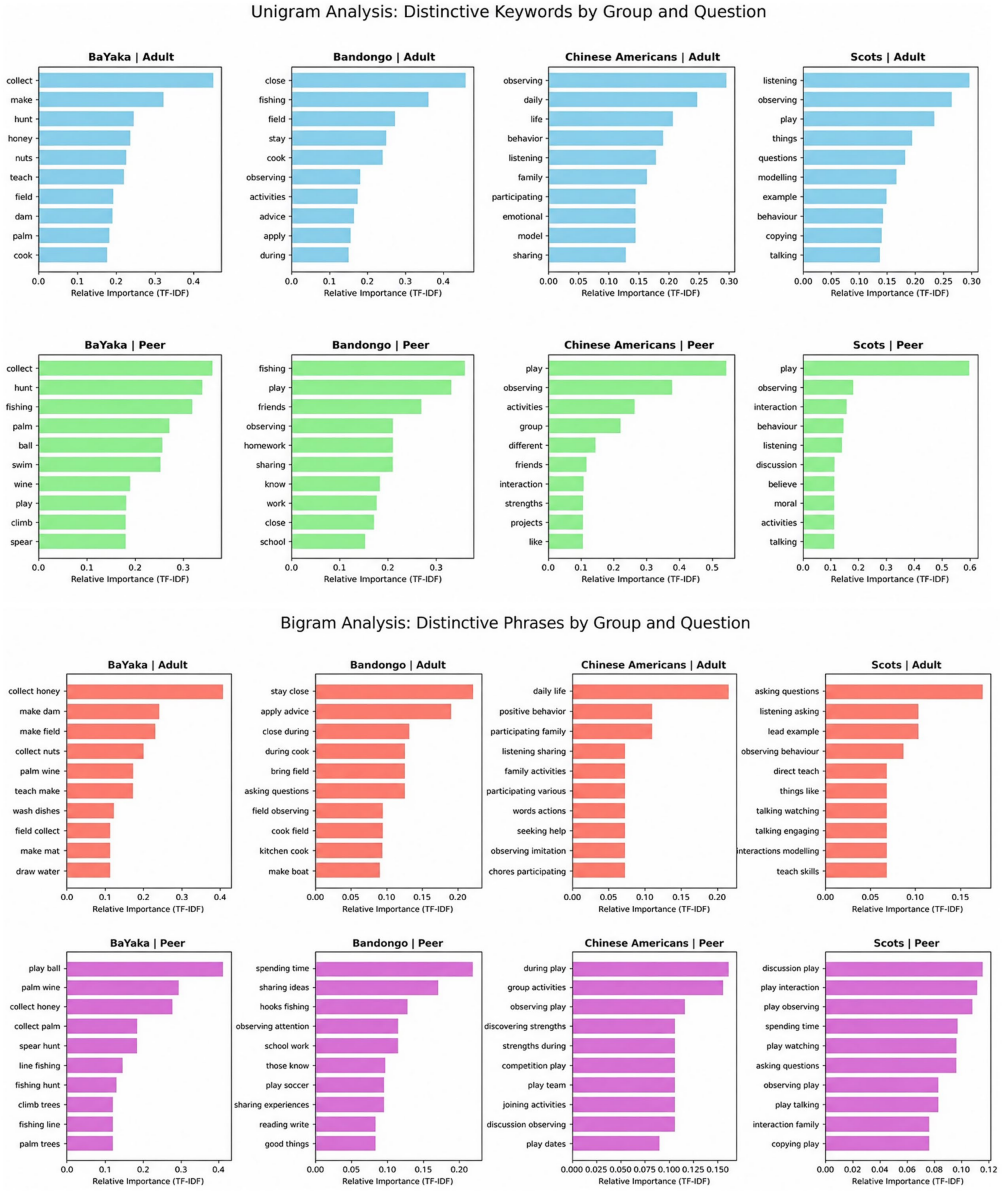
Results from TF-IDF (top) unigram and (bottom) bigram analysis showing distinctive, high-frequency words per target model (peer or adult) and study community.

NLP analysis revealed linguistic patterns of “how” intersecting with “who”, including both similarities and differences across cultures. For example, in TF-IDF unigram analysis (Figure 4, top), “play”, a common collaborative-learning activity among children, was a salient word for peer-focused learning across all four study communities (#1 highest frequency among both Scots and Chinese Americans, #2 highest frequency among Bandongo, and #8 highest-frequency among BaYaka). For adult-focused learning, certain salient words highlighted the importance of other-initiated learning across communities, especially teaching and setting a good example: “modelling” and “example” for Scots, “model” for Chinese Americans, “advice” for Bandongo, and “teach” for BaYaka. Bigram-analysis (Figure 4, bottom) reveals distinctive high-frequency phrases across cultures: for example, “asking questions” appeared in both adult-focused and peer-focused learning for Scots but only in adult-focused learning for Bandongo, and in neither questions for BaYaka and Chinese Americans; in adult-focused learning, “staying close” appeared for Bandongo only, “teach make” (teaching children how to make something) for BaYaka only, and “seeking help” for Chinese Americans only; in peer-focused learning, “competition play” appeared for Chinese Americans only.

## 5 Discussion

Through free-lists regarding what children should learn and open-ended questions regarding how children should learn across four communities, our study investigated parental perspectives on children’s social learning. The central finding is that across all cultures, the source of social learning (who) intersects systematically with both the content of learning (what) and the mechanisms of learning (how). First, across all four communities, adult-focused learning was more frequently characterized by self-initiated learning like active inquiry, intentional knowledge-seeking, or observing/listening/imitation, as well as other-initiated learning like direct teaching, offering guidance/advice/scaffolding, or modeling. In contrast, in peer-focused learning, collaborative learning was consistently more common across the four sites, for example, through play, joint activities, and communication. Echoing Tomasello et al. (1993), this suggests that across cultures, parental expectations of learning pathways and mechanisms are intimately connected, specifically, that children learn *from* adults but *with* peers.

Next, our study revealed culturally specific patterns in the content of social learning, namely, what to learn from adults and what to learn with peers. Types of salient codes differed, with BaYaka and Bandongo focusing more on tasks (school-related knowledge, household and subsistence activities) whereas Scots and Chinese Americans focused more on qualities and values (cooperation, relationships, virtues). The latter two societies are more industrialized and urbanized than the former two, and that is perhaps why domains like household and subsistence activities are more salient for BaYaka and Bandongo. Further, among BaYaka and Bandongo, cooperation (e.g., food sharing, pooled labor) is a central feature of everyday subsistence activities (Boyette and Lew-Levy, 2019; Jang, 2024; Visine et al., 2024; Lew-Levy et al., 2026b); therefore, qualities and values may be assumed to be implicitly transmitted during these tasks. In contrast, scientific parenting discourses, popular among urban middle-class parents in the Global North, increasingly emphasize the explicit transmission of social–emotional competence and wellbeing (Lareau, 2011; Xu, 2017; Zhu et al., 2024), which might explain why Scots and Chinese Americans noted cooperation, relationships, and virtues more than school-related knowledge—at least at a discursive level (as an ideal expressed in survey responses)—and more so compared to BaYaka and Bandongo.

Moreover, what to learn also depends upon whom to learn from/with. In the two Congolese samples, adults were primarily associated with domains tied to responsibility—such as subsistence activities, household tasks, and respect—whereas peers were linked to domains emphasizing participation and shared activity, including athletics, play, and in the Bandongo case certain school-related skills. This distinction reflects both practical reality and cultural models: activities that were usually done with peers were more likely to be learned with peers than from adults. On the other hand, despite children in these communities being active contributors to economic activities, BaYaka and Bandongo parents still viewed adults as key (though not exclusive) transmitters of subsistence and household tasks (see also Lew-Levy et al., 2021). A comparable division emerged among Scots and Chinese Americans, albeit in a different set of domains: adults were associated with life skills, virtues, good behavior, and living well, reflecting the cultural model of adults as the authority figures; peers were associated with cooperation and relationships, aligned with collaborative learning. In this sense, parental models of children’s social learning do not treat “who”, “what,” and “how” as independent dimensions; rather, expectations about the sources and pathways of social learning shape both the kinds of knowledge children are expected to acquire and the processes through which social learning unfolds.

Lastly, salience patterns in the free-list responses might result from the fact that in more industrialized, urbanized communities such as Scots and Chinese Americans there are a greater variety of learning domains than in the BaYaka and Bandongo subsistence context. But a deeper, related factor is that greater global market integration, migration, and population heterogeneity may diffuse consensus in parental expectations about children’s social learning. Parents in the more locally integrated Bandongo and BaYaka communities showed higher convergence in salient items, whereas Chinese American parents in Seattle displayed the most distributed salience patterns. Especially, our Chinese American sample is embedded in a highly heterogeneous, globally connected urban environment, and parents must navigate dual cultural forces (Chinese and American) and adapt to rapid economic and social changes both in their natal country and in the Seattle area.

In summary, the current research investigated parental views regarding the content and mechanisms of social learning from peers and adults. The four study communities shared the perspective that children should learn with other children in a collaborative manner, and learn from adults in a directional manner, initiated by either the learner or by the adult. We also identified between-group variation, namely, culturally specific attitudes in what children should learn as well as concrete expressions about how children should learn. These differences in the “social learning of social learning” (Mesoudi et al., 2016) highlight the reciprocal causation between children’s social learning and their cultural context. Overall, to fully understand how children learn, future research should carefully account for ecological, economic, and social conditions, and consider how parents’ views and children’s development are shaped by both inherited cultural frameworks and emerging lived experience.

## Data Availability

The datasets presented in this study can be found in online repositories. The names of the repository/repositories and accession number(s) can be found at: https://osf.io/wft3c.
