# Supplementary material for: Parental perspectives on children’s social learning in four cultures
Source: Front Psychol. 2026 Jun 11;17:1845262. doi: 10.3389/fpsyg.2026.1845262 (PMC13294349; doi:10.3389/fpsyg.2026.1845262)
Supplement: Supplementary file 1 [file Data_Sheet_1.PDF]

Supplementary Materials for:
Parental perspectives on children's social learning in four cultures

Free list coding

Art: Engagement in creative and expressive artistic practices that involve performance, rhythm, movement, or visual creation. Examples: singing, dancing, drumming, drawing.

Communication: The ability to exchange meaning with others through verbal and non-verbal interaction, including speaking, listening, explaining, negotiating, sharing feelings, humour, asking for help, giving advice, and managing conversations or disagreements.

Cooperation: Working with others in a mutually supportive way to achieve shared goals, including teamwork, compromise, helping, turn-taking, collaboration, leadership, fairness, and maintaining positive group relations. *N.B.* topics related to connection, friendship, or interpersonal closeness were coded as 'Relationships' (see below).

Curiosity: A desire to learn, explore, and discover new knowledge, skills, experiences, or perspectives through questioning, experimentation, interest, and motivation to try new things.

Diversity: Recognition, acceptance, and appreciation of differences in people, perspectives, identities, and ways of living, including tolerance and inclusion.

Emotions: Awareness, expression, and regulation of feelings in oneself and others, including managing reactions, understanding sadness or joy, and emotional self-control.

Family Values & Cultural Traditions: Knowledge and practice of shared cultural beliefs, customs, history, and behaviors transmitted within families and communities.

Good Behavior: Acting according to social expectations and norms, including manners, etiquette, appropriate conduct, discipline, and learning from (social) mistakes.

Household: Practical domestic responsibilities required to maintain a home, such as cleaning, cooking, repairing, building, and completing daily chores.

Life Skills: Practical competencies for functioning effectively in everyday life, including planning, organization, problem management, independence, transferable skills, and real-world know-how beyond academics.

Living Well: Understanding how to live a meaningful, balanced, and fulfilling life, including wellbeing, happiness, priorities, habits, and the value of life. Items related to self-care and hygiene are excluded (see Self-Care).

Money: Understanding and managing financial resources, including earning, budgeting, and financial responsibility.

Obedience: Following rules, routines, authority, and expected roles or responsibilities within structured settings.

Occupation: Knowledge or preparation for work, trades, professions, or economic activities, including technical and vocational skills. For Congo, this includes preparing items for sale (e.g., baking bread, making donuts).

Old & New Media: Awareness and use of information sources and technologies, including books, digital tools, and contemporary trends.

Play & Fun: Participation in enjoyable recreational activities undertaken for pleasure, entertainment, and social enjoyment.

Relationships: Forming, maintaining, and navigating interpersonal connections, including friendship, family roles, boundaries, support, and social interaction. *N.B.* topics related to shared task or joint activity were coded as 'Cooperation' (see above).

Religion: Beliefs and practices related to spirituality or faith, including prayer, worship, and respect toward a deity.

Resilience: The capacity to cope with challenges, persist through difficulty, take risks, and recover from setbacks through effort and determination.

Respect: Showing consideration, regard, and appropriate treatment toward oneself and others.

School-Related: Academic learning and formal education skills such as literacy, numeracy, studying, and subject knowledge.

Self-Care: Personal health, hygiene, bodily care, and safety practices that support independence and wellbeing.

Athletics: Participation in structured physical activities involving exercise, skill, or competition.

Subsistence: Activities that support survival and food provision, such as farming, fishing, hunting, gathering, and animal keeping.

Virtues: Moral qualities and positive character traits guiding ethical behaviour, such as compassion, kindness, honestly, integrity, independence, love, loyalty.

Wisdom & Intelligence: Cognitive abilities used to understand, reason, evaluate, and make informed decisions, including critical thinking, risk assessment, creativity, and judgement.

Not Coded: Content that is unclear, irrelevant, too vague, or does not fit any defined categor

Supplementary Tables

Table S1. Results from the free-list salience analysis. Values closer to 1 represent higher salience, i.e., how often and how participants reported the item.

	Bandongo		BaYaka		Scots		Chinese Americans	
CODE	Adult	Peer	Adult	Peer	Adult	Peer	Adult	Peer
Art	--	0.0306	0.0119	0.0221	--	--	--	0.0056
Athletics	0.0360	0.1643	0.0017	0.6865	0.0024	0.0222	--	0.0215
Communication	0.0901	0.0330	--	--	0.2089	0.1892	0.0449	0.2044
Cooperation	0.0734	0.0325	--	0.0051	0.1528	0.4258	0.0307	0.3768
Curiosity	0.0114	--	--	--	0.0489	0.1052	0.0859	0.1634
Diversity	--	--	--	--	0.0563	0.1149	0.0038	0.0719
Emotions	--	--	--	--	0.0889	0.0177	0.0753	0.0114
Family Values & Cultural Traditions	0.0099	0.0081	--	--	0.0197	0.0131	0.0361	0.0167
Good behaviour	0.0540	0.0408	0.0085	--	0.2856	0.0981	0.1048	0.1209
Household	0.4095	0.3182	0.5970	0.1343	0.0558	--	0.0481	--
Life Skills	--	--	--	--	0.2722	0.0877	0.2656	0.1212
Living Well	0.0598	0.0045	0.0149	--	0.0305	--	0.2104	0.0490
Money	0.0023	--	--	--	0.0318	--	0.0112	--
Obedience	0.0076	0.0014	--	--	0.0812	0.0267	0.0179	--
Occupation	0.0964	0.0851	--	--	--	--	0.0096	--
Old & New Media	--	--	--	--	0.0022	0.0037	--	0.0131
Play & Fun	0.0028	0.2602	0.0200	0.2410	0.0476	0.1862	0.0144	0.0474
Relationships	0.0147	0.0045	--	0.0187	0.2389	0.4096	0.0481	0.2239
Religion	0.0519	0.0911	--	--	0.0140	--	--	--
Resilience	--	--	--	--	0.0746	0.0612	0.1923	0.0431
Respect	0.2605	0.1234	--	--	0.2038	0.0812	0.0669	0.0333
School-Related	0.2790	0.6395	0.0377	0.0811	0.0767	0.0252	0.0240	0.0067
Self-Care	0.0284	0.0476	0.0112	0.0212	0.0881	0.0110	0.1049	0.0343
Subsistence	0.5692	0.3665	0.7902	0.7714	--	--	--	--
Virtues	0.0085	0.0304	--	--	0.5263	0.3196	0.1639	0.1732
Wisdom & Intelligence	0.0553	0.1558	--	--	0.1866	0.0882	0.2830	0.0752

Table S2. Results from the multilevel logistic regression investigating the presence/absence of each learning category (self-initiated, other-initiated, collaborative) by learning model (peer, adult).

Variable	Estimate	Est.Error	l-95% CI	u-95% CI
Intercept	-0.552	0.191	-0.923	-0.179
Peer	1.643	0.277	1.118	2.202
Learner-initiated	1.854	0.317	1.242	2.482
Other-initiated	0.427	0.303	-0.183	1.015
BaYaka	-1.031	0.322	-1.670	-0.415
Scots	0.801	0.267	0.278	1.327
Chinese Americans	0.989	0.308	0.394	1.598
Peer X Learner-initiated	-2.314	0.404	-3.137	-1.542
Peer X Other-initiated	-4.633	0.499	-5.652	-3.697
Peer X BaYaka	1.940	0.433	1.101	2.797
Peer X Scots	0.199	0.394	-0.565	0.983
Peer X Chinese Americans	0.131	0.454	-0.748	1.029
Learner-initiated X BaYaka	-2.028	0.535	-3.100	-1.005
Other-initiated X BaYaka	2.791	0.498	1.834	3.794
Learner-initiated X Scots	-1.581	0.436	-2.432	-0.718
Other-initiated X Scots	-0.953	0.438	-1.825	-0.099
Learner-initiated X Chinese Americans	-0.634	0.525	-1.657	0.432
Other-initiated X Chinese Americans	-1.561	0.494	-2.539	-0.600
Peer X Learner-initiated X BaYaka	-2.808	0.681	-4.161	-1.504
Peer X Other-initiated X BaYaka	-1.987	0.605	-3.201	-0.812
Peer X Learner-initiated X Scots	-0.355	0.551	-1.454	0.717
Peer X Other-initiated X Scots	-0.366	0.626	-1.610	0.858
Peer X Learner-initiated X Chinese Americans	-1.265	0.625	-2.504	-0.044
Peer X Other-initiated X Chinese Americans	-0.481	0.728	-1.925	0.928

Table S3. Contrasts (adult — peer) and 95% Highest Posterior Density Intervals (HPDI) for presence/absence of each learning category (self-initiated, other-initiated, collaborative) by study community. Values in bold are those for whom HPDI intervals do not cross 0.

Site	Code	Median	lower.HPD	upper.HPD

Bandongo	Collaborative	**-0.380**	**-0.486**	**-0.267**
Bandongo	Learner-initiated	0.131	-0.003	0.275
Bandongo	Other-initiated	**0.422**	**0.304**	**0.545**
BaYaka	Collaborative	**-0.704**	**-0.798**	**-0.603**
BaYaka	Learner-initiated	**0.108**	**0.020**	**0.217**
BaYaka	Other-initiated	**0.628**	**0.471**	**0.769**
Scots	Collaborative	**-0.325**	**-0.435**	**-0.213**
Scots	Learner-initiated	**0.198**	**0.019**	**0.384**
Scots	Other-initiated	**0.395**	**0.261**	**0.538**
Chinese Americans	Collaborative	**-0.288**	**-0.424**	**-0.160**
Chinese Americans	Learner-initiated	**0.369**	**0.164**	**0.566**
Chinese Americans	Other-initiated	**0.311**	**0.165**	**0.475**
